# MicroRNA Dynamics and Functions During Arabidopsis Embryogenesis

**DOI:** 10.1105/tpc.19.00395

**Published:** 2019-09-27

**Authors:** Alexandra Plotnikova, Max J. Kellner, Michael A. Schon, Magdalena Mosiolek, Michael D. Nodine

**Affiliations:** Gregor Mendel Institute, Austrian Academy of Sciences, Vienna Biocenter, 1030 Vienna, Austria

## Abstract

MicroRNAs (miRNAs) are short noncoding RNAs that mediate the repression of target transcripts in plants and animals. Although miRNAs are required throughout plant development, relatively little is known regarding their embryonic functions. To systematically characterize embryonic miRNAs in Arabidopsis (*Arabidopsis thaliana*), we developed or applied high-throughput sequencing-based methods to profile hundreds of miRNAs and associated targets throughout embryogenesis. We discovered dozens of miRNAs that dynamically cleave and repress target transcripts, including 30 that encode transcription factors. Transcriptome analyses indicated that these miRNA:target interactions have profound effects on embryonic gene expression programs. Moreover, we demonstrated that the miRNA-mediated repression of six transcription factors are individually required for proper division patterns of various embryonic cell lineages. These data indicate that the miRNA-directed repression of multiple transcription factors is critically important for the establishment of the plant body plan, and they provide a foundation to further investigate how miRNAs contribute to these initial cellular differentiation events.

## Abstract (precis)

Genome-wide analysis of microRNA dynamics and functions during *Arabidopsis thaliana* embryogenesis uncovers microRNA:target interactions with profound effects on embryonic gene expression and morphogenesis programs.

## INTRODUCTION

MicroRNAs (miRNAs) are a class of small regulatory RNAs (sRNAs) that posttranscriptionally repress gene expression and regulate cellular differentiation during plant and animal development (Bartel, 2004; Jones-Rhoades et al., 2006; Plasterk, 2006; Chen, 2009). Plant miRNA precursors fold into characteristic RNA stem-loop structures that are recognized and processed into mature ∼21-nucleotide miRNAs by the *RNa*seIII domain-containing protein DICER-LIKE1 (DCL1; Park et al., 2002; Reinhart et al., 2002). miRNAs are then loaded onto ARGONAUTE1 (AGO1) proteins and guide the complex to sequences in target RNAs that are almost perfectly complementary to the miRNA (Jones-Rhoades and Bartel, 2004; Allen et al., 2005). In general, miRNAs recognize single sites in target transcripts, and the high degree of miRNA:target duplex basepairing results in target RNA cleavage, although translational repression has also been reported (Llave et al., 2002; Aukerman and Sakai, 2003; Kasschau et al., 2003; Chen, 2004; Jones-Rhoades and Bartel, 2004; Gandikota et al., 2007). The miRNA-mediated cleavage and repression of transcripts including those encoding transcription factors is required throughout development (Jones-Rhoades et al., 2006; Chen, 2009; D’Ario et al., 2017).

Although miRNAs have been implicated in an array of postembryonic developmental processes, their functions during embryogenesis remain less well-characterized (Vashisht and Nodine, 2014). This is primarily due to early embryos being small and deeply embedded in maternal seed coat tissues, which makes it difficult to isolate them at high purity and characterize the corresponding RNA populations (Schon and Nodine, 2017). Nevertheless, the precursors of the shoot and root meristems and three main radial tissue layers are precisely established during early embryogenesis, and miRNAs are required for most of these early patterning events (Schwartz et al., 1994; Nodine and Bartel, 2010; Willmann et al., 2011; Seefried et al., 2014). Moreover, miRNAs are required to prevent the precocious expression of genes involved in embryo maturation when storage macromolecules such as oil bodies accumulate (Nodine and Bartel, 2010; Willmann et al., 2011). Embryonic miRNAs therefore help define cell-specific gene expression programs according to both spatial and temporal cues. For example, miR165/166 spatially restrict RNAs encoding homeobox-leucine zipper family transcription factors during embryogenesis (McConnell et al., 2001; Smith and Long, 2010; Miyashima et al., 2013), and miR156/157-mediated repression of SQUAMOSA PROMOTER BINDING PROTEIN-LIKE (SPL) transcription factor genes is required for both the proper divisions of root meristem precursors and to prevent the precocious expression of maturation phase genes (Nodine and Bartel, 2010). Arabidopsis (*Arabidopsis thaliana*) *mir160a* loss-of-function mutant embryos divide incorrectly, and the abnormal cotyledon phenotypes of seedlings expressing transgenes containing mutations in miR160, miR170/171, or miR319 target sites suggest that the corresponding miRNA activities are required for embryo morphogenesis (Palatnik et al., 2003; Mallory et al., 2005; Liu et al., 2010; Takanashi et al., 2018). The cell-type–specific miR394-mediated repression of transcripts encoding the LCR F-box protein is also required for patterning embryonic apical domains (Knauer et al., 2013).

Despite these individual examples of embryonic miRNA functions and miRNA profiling studies on late-staged plant embryos (Oh et al., 2008; Huang et al., 2013; Xu et al., 2018), a comprehensive understanding of embryonic miRNA populations and their individual contributions to embryogenesis is incomplete. Arabidopsis embryos are ideal model systems to investigate the roles of miRNAs during plant embryogenesis. Not only do the available genomic and genetic resources in Arabidopsis facilitate the functional characterization of miRNA, but Arabidopsis embryos undergo a series of highly stereotypical cell divisions to generate the basic body plan (Mansfield and Briarty, 1991; Palovaara et al., 2016). Therefore, early Arabidopsis embryos with disrupted miRNA functions can be examined for abnormal cell division patterns to test whether the corresponding miRNAs are required for morphogenesis, and thus yield insights into the molecular basis of the corresponding patterning events.

In this study, we developed a low-input small RNA sequencing (sRNA-seq) method to generate profiles of hundreds of miRNAs and used the recently developed parallel analysis of RNA 5′ ends (nanoPARE) approach (Schon et al., 2018) to identify corresponding target transcripts throughout embryogenesis. We found that miRNAs dynamically cleave and repress at least 59 transcripts, including 30 encoding transcription factors belonging to eight different families. As a proof-of-principle of this data set’s utility, we selected individual miRNA/target interactions to investigate further and demonstrated that the miRNA-mediated repression of six RNAs encoding transcription factors are individually required for the proper cell division patterns of various postembryonic tissue-type precursors. Therefore, this resource provides a foundation to further investigate how miRNAs help coordinate the formation of the basic body plan by posttranscriptionally restricting their targets, including transcription factors, to specific stages and cell-types.

## RESULTS

### Establishment of Low-Input sRNA-Seq Method

To systematically characterize the dynamics and functions of individual embryonic miRNAs in Arabidopsis, it was first necessary to identify the miRNAs present in developing embryos. However, standard high-throughput sRNA-seq methods require relatively large amounts of total RNA, which are impractical to obtain from early embryos. The sequential ligation of adapters onto the hydroxyl and monophosphate groups at the respective 3′ and 5′ termini of sRNAs, followed by reverse transcription and PCR amplification during conventional sRNA-seq library preparation, requires ≥500 nanograms (ng) of total RNA, which is ∼100 times more than can be obtained from early Arabidopsis embryos. More recent sRNA-seq methods can profile sRNAs from as little material as a single cell, but they do not enrich for sRNAs to the same extent as conventional methods (Faridani et al., 2016). Therefore, to enable the profiling of miRNAs present in developing Arabidopsis embryos, we developed a method employing the NEBNext Multiplex Small RNA Library Prep Set for Illumina Kit (New England Biolabs) that is suitable for the low amounts of total RNA obtainable from early embryos (i.e. 1 to 5 ng). In brief, we included polyacrylamide gel-based size-selection methods to enrich for sRNAs from total RNA before the first adapter ligation step, as well as to enrich for desired sRNA cDNAs after final PCR amplification (see Methods for details). We also reduced the amounts of 3′ adapters, reverse transcriptase primers, and 5′ adapters used in the NEBNext kit when starting with ≤500 ng of total RNA.

**Figure 1.**
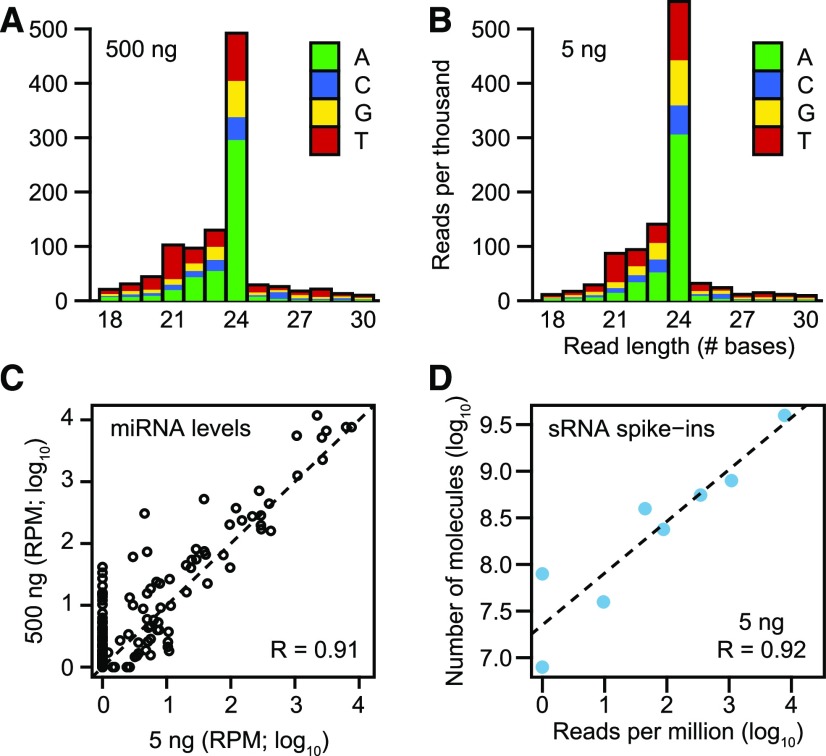
Establishment of Low-Input sRNA-Seq Method **(A)** and **(B)** Stacked bar charts of normalized sRNA-seq read levels (reads per thousand genome-mapping reads) across different base lengths in libraries generated with either 500 ng **(A)** or 5 ng **(B)** of total RNA isolated from bent-cotyledon stage embryos. Colors indicate the proportions of sRNA-seq reads that begin with various bases as indicated in the key. **(C)** Scatterplot of miRNA family levels in sRNA-seq libraries generated from 5 ng and 500 ng of total RNA. sRNA levels were normalized for RPM and log_10_-transformed. Pearson’s *R* value is indicated, as well as a dashed line with an intercept of 0 and slope of 1. **(D)** Scatterplot of relative sRNA spike-in levels (RPM; log_10_) as compared with the absolute number of sRNA spike-in molecules (log_10_) added during RNA isolation for a sRNA-seq library generated from 5 ng of total RNA. Pearson’s *R* value is shown, and the dashed line represents a linear model derived from the plotted data points.

We compared sequencing data from libraries generated with 500, 50, 5, 1, or 0.5 ng of total RNA isolated from bent-cotyledon stage Columbia-0 (Col-0, hereafter referred to as “wild-type”) embryos to determine how well the method enriches for sRNAs, as well as the method’s reproducibility and accuracy when starting with different amounts of total RNA. Approximately 21-nucleotide miRNAs and 24-nucleotide small interfering RNAs that typically begin with uridine- and adenosine-monophosphates, respectively, are characteristic features of plant sRNA populations (Borges and Martienssen, 2015). As expected for plant sRNAs, libraries generated from all input amounts of total RNA predominantly consisted of 21- to 24-base reads, with the first position of the 21- and 24-base reads enriched for thymine and adenine, respectively (Figures 1A and 1B; Supplemental Figures 1A to 1C). The distribution of sRNA-seq read sizes and 5′ nt biases indicated that the sRNA-seq protocol highly enriches for sRNAs from as little as 0.5 ng of total RNA. To determine the reproducibility of the method across various amounts of input RNA, we compared miRNA family levels between libraries constructed from 500 ng of total RNA with those generated from either 50, 5, 1, or 0.5 ng of total RNA. miRNA levels were highly correlated between biological replicate libraries generated from 500 ng of total RNA (Pearson’s *R* > 0.99; Supplemental Figures 1D and 1E). Pearson’s correlation coefficients were >0.9 between 500-ng libraries and all libraries generated from ≥1 ng of total RNA (Figure 1C; Supplemental Figures 1F to 1H).

We also assessed the accuracy of this low-input sRNA-seq method across the dilution series of input RNA by adding exogenous sRNA oligonucleotides (i.e. spike-ins; Lutzmayer et al., 2017) during RNA isolation before library construction and examined spike-in levels in the resulting sRNA-seq data sets. If the method accurately quantified sRNA levels, we would expect a high correlation between the absolute number of spike-in molecules added and the number of sRNA-seq reads mapping to the spike-ins. Pearson’s correlation coefficients between the absolute amounts of spike-ins added and the relative amounts of spike-ins sequenced were >0.9 for all libraries generated from ≥1 ng total RNA (Figure 1D; Supplemental Figures 1I to 1L). The progressive increase in the number of undetected miRNA families and sRNA spike-ins as total RNA amounts decreased indicated that the sensitivity of the method was reduced when starting with <50 ng of total RNA (Figures 1C and 1D; Supplemental Figures 1G, 1H, 1K, and 1L). Regardless, the modified sRNA-seq library construction method allowed us to highly enrich for sRNAs and to reproducibly and accurately quantify miRNA levels when starting with 1–5 ng of total RNA, which are amounts obtainable from early Arabidopsis embryos.

### Embryonic miRNA Dynamics

**Figure 2.**
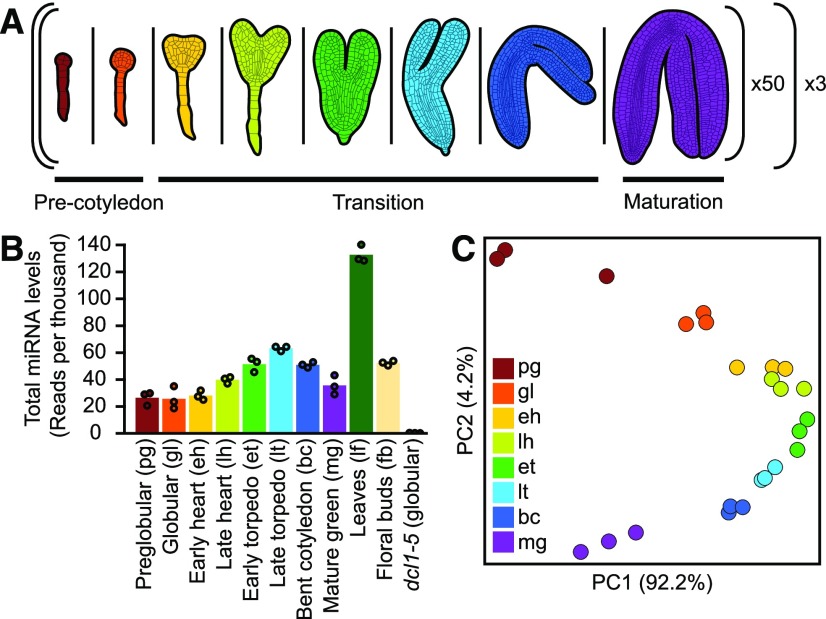
Application of the Low-Input sRNA-Seq Method to Arabidopsis Embryos **(A)** Schematic of sRNA profiling experiment across embryogenesis with the low-input sRNA-seq method. Fifty embryos from each of eight different embryonic stages spanning three indicated main phases of embryogenesis were pooled into individual biological replicates. This was repeated three times for each stage to generate three biological replicates for each of the eight developmental stages (i.e. 24 libraries from a total of 1,200 embryos). **(B)** Bar chart displaying the total amount of miRNAs detected across wild-type embryogenesis, leaves, and floral buds, as well as in miRNA-deficient *dcl1*-*5* embryos isolated at the globular stage. miRNA levels were normalized by reads per thousand genome-mapping reads. Points indicate the mean levels of individual biological replicates. Individual stages together with their abbreviations are labeled. **(C)** Principal component analysis illustrating the relationships of the 24 sRNA-seq libraries generated from wild-type embryonic tissues based on miRNA levels. Embryonic stages are labeled according to the key.

We then used this low-input sRNA-seq method to generate libraries using total RNA isolated from embryos at eight developmental stages including three main phases of embryogenesis (Figure 2A; Supplemental Data Set 1; Hofmann et al., 2019). Three pools of 50 embryos were isolated from each of the eight stages from different plants and on different days, and considered biological replicates (1,200 embryos in total). At least 80% of the total RNA isolated from each biological replicate was used to generate sRNA-seq libraries, and the remainder was used to generate full-length cDNAs to profile either transcriptomes (Hofmann et al., 2019) or miRNA-mediated cleavage products. Previous analysis of mRNA-seq libraries generated from an aliquot of the same total RNA demonstrated that the embryonic RNA samples were not significantly contaminated with nonembryonic RNAs (Hofmann et al., 2019), which had been a frequent problem in early embryonic Arabidopsis transcriptome data sets (Schon and Nodine, 2017; Hofmann et al., 2019). Total miRNA levels fluctuated in wild-type embryos according to their developmental stage, but were almost completely lost in *dcl1*-5 null mutants (Figure 2B). Because *DCL1* is required for miRNA biogenesis (Park et al., 2002; Reinhart et al., 2002), this further supports the validity of the miRNAs identified in the sRNA-seq libraries. Principal component analysis of miRNA family levels in libraries generated from embryonic and postembryonic tissues demonstrated that biological replicates clustered together (Figure 2C; Supplemental Figure 2). Furthermore, the developmental stages of the embryonic samples were stratified along the second principal component and were clearly separated from the postembryonic leaf and flower samples. By applying the low-input sRNA-seq method to developing embryos, we were therefore able to generate high-quality profiles of embryonic miRNAs, which changed in composition across developmental stages.

**Figure 3.**
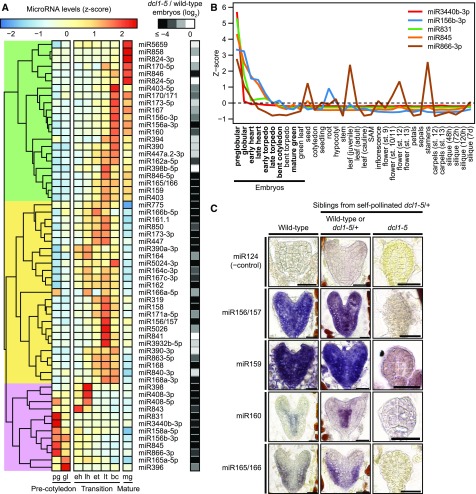
miRNA Dynamics During Embryogenesis **(A)** Heat map illustrating the relative levels of miRNA families across embryogenesis. miRNA families with ≥10 mean RPM in at least one embryonic stage are shown, and colors represent z-scores for each individual miRNA family according to the key. Log_2_-transformed levels of miRNAs in (*dcl1*-*5* + 1)/(wild-type + 1) are annotated. Three major phases of embryo development are labeled at the bottom, and individual columns are labeled according to stage: pg = preglobular; gl = globular; eh = early heart; lh = late heart; et = early torpedo; lt = late torpedo; bc = bent cotyledon; mg = mature green. The dendrogram is highlighted in green, yellow, or violet to indicate three clusters of miRNA families. **(B)** Line graph depicting relative levels (z-scores) of preglobular-enriched miRNA families across development. Five miRNA families were selected based on their enrichment in embryos as compared with internally generated leaf and floral bud sRNA-seq libraries. sRNA-seq libraries generated internally are marked in bold, and published sRNA-seq data from 26 tissue types (Xu et al., 2018) are also shown. **(C)** Representative images of miRNA in situ hybridizations on sections of embryos. Probes antisense to four miRNAs detected in embryonic sRNA-seq libraries are shown, and the corresponding miRNA families are labeled. Probes antisense to the mouse miR124 were used as negative controls, as well as miRNA-deficient *dcl1*-*5* embryos. Scale bars = 20 µm.

We detected 349 miRNAs belonging to 259 families in at least one embryonic stage (Supplemental Data Set 2). We then selected 59 miRNA families detected with an average of ≥10 reads per million genome-matching reads (RPM) in at least one embryonic stage to examine in greater detail. Three groups of miRNAs with similar dynamics across embryogenesis were observed (Figure 3A; Supplemental Figure 3). Twenty-two miRNA families accumulated during the late transition phase and persisted in mature green embryos. These included miR394, miR403, and miR170/171, as well as miR167 and miR390, which were both previously detected in late-stage embryos with whole-mount RNA in situ hybridizations (Ghosh Dastidar et al., 2016). Another set of 25 miRNA families, including miR156/157, miR161, miR164, and miR319, accumulated during the transition phase, but their levels were then reduced in mature embryos. Twelve miRNA families had relatively high levels during early embryogenesis and decreased thereafter. Based on further analysis of internally generated and publicly available sRNA-seq data from 26 tissue types (Xu et al., 2018), five miRNA families were highly enriched during the initial stages of embryogenesis, including miR156b-3p, miR831, miR845, miR866-3p, and miR3440b-3p (Figure 3B; Supplemental Figures 4).

To examine whether miRNA levels vary between early embryonic cell types, we adapted a whole-mount sRNA in situ protocol (Ghosh Dastidar et al., 2016) to detect four selected miRNAs in sections of early embryos. Consistent with previous reports (Nodine and Bartel, 2010), miR156/157 was localized throughout wild-type embryos, and a similar pattern was also observed for miR159 (Figure 3C). miR165/166 confers repressive activities in the peripheral cell-types of embryos (McConnell et al., 2001; Smith and Long, 2010; Miyashima et al., 2013), and miR165/166 levels were accordingly higher in these outer cell types (Figure 3C). By contrast, miR160 levels were higher in the innermost vascular precursor cells (Figure 3C). sRNA in situs performed with probes antisense to the mouse-specific miR124 miRNA produced low signal as compared with probes antisense to the four miRNAs in wild-type embryos or embryos with wild-type morphologies from *dcl1*-*5*/+ self-pollinated plants (i.e. wild-type or *dcl1*-*5*/+ embryos; Figure 3C). Moreover, probes antisense to the four miRNAs produced highly reduced signals when applied to miRNA-deficient *dcl1*-*5* embryos as compared with wild-type or *dcl1*-*5*/+ embryos (Figure 3C). These controls further support the specificity of the signal observed from the miRNA in situ hybridizations.

### Identification of Embryonic miRNA Targets

Based on our analyses, embryonic miRNA populations were distinct from those in postembryonic tissues, and their levels frequently exhibited dynamic changes across developmental stages and sometimes cell types. These results suggest that miRNAs have distinct functions during different phases of embryogenesis. Because miRNA functions are largely defined by the targets they regulate, we next determined the targets of embryonic miRNAs. In plants, miRNAs typically bind to highly complementary binding sites within target RNAs and mediate their endonucleolytic cleavage (Llave et al., 2002; Kasschau et al., 2003; Jones-Rhoades and Bartel, 2004). miRNA-mediated cleavage of target RNAs produces cleavage products downstream of the slice site, which can be cloned and sequenced with high-throughput methods referred to as “parallel analysis of RNA ends” (PARE), “genome-wide mapping of uncapped and cleaved transcripts,” or “degradome sequencing” (Addo-Quaye et al., 2008; German et al., 2008; Gregory et al., 2008). Although these groundbreaking technologies have allowed miRNA target identification on a genome-wide scale, they require ≥10,000-fold more input RNA than what was obtainable from early Arabidopsis embryos. We previously developed a method called “nanoPARE” to enable the confident identification of miRNA-mediated target RNA cleavage products from low-input RNA (Schon et al., 2018). To identify embryonic miRNA targets, we therefore generated nanoPARE libraries from the same eight stages of embryogenesis used for miRNA profiling in biological triplicates. In addition to these 24 libraries from wild-type embryos, we also generated nanoPARE libraries from three biological replicates of *dcl1*-*5* globular embryos as controls (Supplemental Data Set 1).

**Figure 4.**
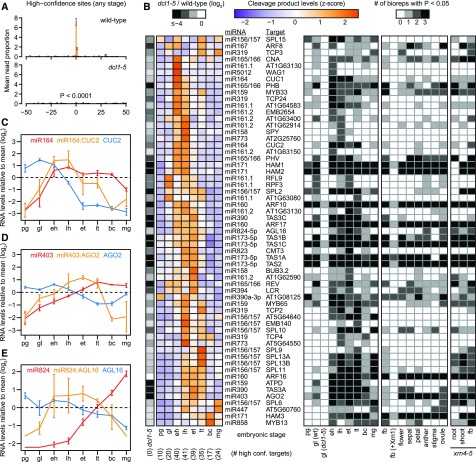
Identification of Embryonic miRNA Targets **(A)** The proportion of nanoPARE reads mapping within 50-nucleotides of miRNA target sites significantly detected by “EndCut” (Benjamini–Hochberg-adjusted *P* values < 0.05) in ≥2 biological replicates from any embryonic stage (i.e. high-confidence miRNA cleavage sites; *n* = 59) are shown for wild-type (*top*) and *dcl1*-*5* (*bottom*) globular embryo libraries. The probability (*P* value) that the mean number of reads at the predicted cleavage sites in *dcl1*-*5* is different from the wild-type mean due to chance is indicated (two-tailed K–S test). Error bars represent mean ± se of three biological replicates. **(B)** Heat maps depicting the relative levels of miRNA-mediated cleavage products (*left*) and the number of biological replicate libraries in which cleavage products were significantly detected (*right*). High-confidence miRNA cleavage sites from any embryonic stage are shown together with miRNA families and target transcripts alongside the rows. Colors represent z-scores as indicated in the key. Log_2_-transformed levels of cleavage products in (*dcl1*-*5* + 1)/(wild-type + 1) are annotated, and embryonic stages are indicated beneath each column, as well as the number of high-confidence targets from that stage. (*right*) Shading densities in the heatmap on the right indicate the number of biological replicates for which the cleavage site was significantly detected by the tool “EndCut” according to the key (Benjamini–Hochberg-adjusted *P* value < 0.05). Embryonic stages and postembryonic tissues in wild-type and *xrn4*-*5* genotypes are indicated beneath each column. *dcl1*-*5* = *dcl1*-*5* globular; pg = preglobular; gl = globular; eh = early heart; lh = late heart; et = early torpedo; lt = late torpedo; bc = bent cotyledon; mg = mature green; fb = unopened floral buds; +Xrn1 = RNA treated with Xrn1 exoribonuclease before library construction. **(C to E)** Line graphs illustrating the relative RNA levels of miRNAs (red), targets (blue), and miRNA-mediated cleavage products (orange) for miR164:CUP-SHAPED COTYLEDON2 **(C)**, miR403:AGO2 **(D)** and miR824:AGAMOUS-LIKE16 **(E)**. Error bars represent mean ± se of three biological replicates for each stage. Relative levels of miRNAs (RPM), cleavage products (reads per ten million genome-matching reads) and transcripts (TPM) for each stage were calculated by log_2_-transforming (stage levels + 1)/(mean levels across all stages + 1). Embryonic stage abbreviations beneath each graph are as indicated in **(B)**.

The nanoPARE data sets and target predictions for 164 miRNAs detected ≥1 RPM in ≥1 embryonic stage were used as input for the software “EndCut” (Schon et al., 2018). We identified 115 significant target transcript cleavage sites in ≥1 embryonic library (Benjamini–Hochberg-adjusted *P* values < 0.05; Supplemental Data Set 3). These 115 target sites included 59 sites that were identified in ≥2 biological replicates from ≥1 developmental stage. We refer to these as “high-confidence” targets, and characterized these 59 sites corresponding to 22 miRNA families further. The first positions of nanoPARE reads mark RNA 5′ ends. The number of nanoPARE reads at the 59 high-confidence target sites detected in wild-type embryos was significantly reduced (40.5-fold) in miRNA-deficient *dcl1*-*5* globular embryos (*P* value < 0.0001; two-tailed K-S test; Figure 4A). Moreover, no high-confidence targets were detected in *dcl1*-*5* embryos, and 58/59 of the high-confidence targets detected in developing wild-type embryos had decreased numbers of nanoPARE reads in *dcl1*-*5* embryos (Figure 4B). A lack of signal in *dcl1*-*5* embryos could be explained by either a loss of miRNA-mediated cleavage or technical differences in sample RNA quality. To differentiate between these two explanations, we measured nanoPARE signal mapping to published transcription start sites (Schon et al., 2018) of all high-confidence targets detected in globular embryos. Full-length transcripts were more abundant in *dcl1*-*5* embryos for 17/20 of these high-confidence targets, demonstrating that the observed reduction of nanoPARE signal at miRNA-directed cleavage sites in *dcl1*-*5* embryos was not due to differences in RNA quality (Supplemental Figure 5). The loss of miRNA-mediated cleavage sites in miRNA-deficient *dcl1*-*5* embryos further supports the validity of the miRNA targets identified.

miRNA-mediated cleavage products dynamically accumulated and were generally more abundantly detected during mid-embryogenesis (Figure 4B). To identify miRNA-mediated cleavage events that are enriched in embryos, we also analyzed nanoPARE libraries generated either previously from flowers and floral organs (Schon et al., 2018), or in this study from root or shoot tissues of *exoribonuclease4*-*5* (*xrn4*-*5*) mutants, in which miRNA-directed cleavage products are stabilized (Souret et al., 2004; German et al., 2008). We observed 11 high-confidence target transcripts enriched in developing embryos, including those encoding the EMB2654 (miR161.2) and SPINDLY (miR158) tetratricopeptide repeat proteins involved in embryogenesis and gibberellic acid responses, respectively; an ATP synthase delta subunit (ATPD; miR159); a plant invertase/methylesterase inhibitor family protein (AT5G64640; miR156/157); and TCP4 and TCP24 (miR319) transcription factors. Interestingly, a simple linear relationship between miRNA abundance and cleavage products was sufficient to explain the dynamics of only a minority of the observed miRNA/target level dynamics during embryogenesis (Supplemental Figure 6). However, a few miRNA/target cleavage products accumulated during embryonic stages at which miRNA levels were increasing and full-length target transcripts were decreasing. For example, miR164:CUP-SHAPED COTYLEDON2 and miR824:AGAMOUS-LIKE16 cleavage products accumulated during mid-embryogenesis when miRNA and target levels were increasing and decreasing, respectively (Figures 4C to 4E). Similarly, miR403:AGO2 products were present at relatively high levels during midembryogenesis when increasing miR403 levels were concomitant with decreasing AGO2 levels (Figure 4D).

### Impact of miRNAs on the Embryonic Transcriptome

**Figure 5.**
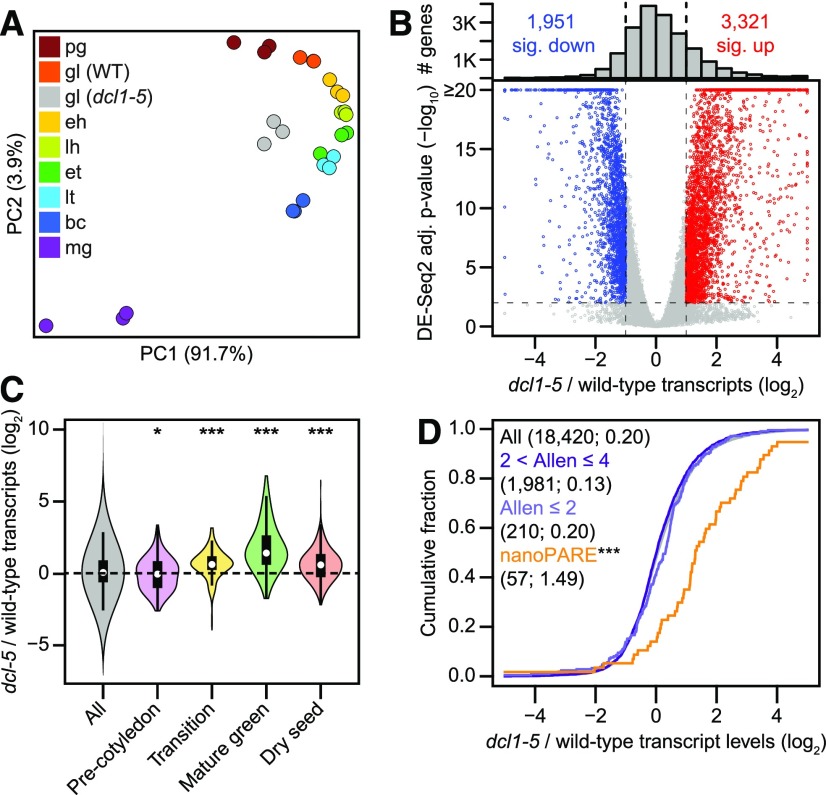
Impact of miRNAs on the Embryonic Transcriptome **(A)** Principal component analysis illustrating the relationships between mRNA-seq libraries generated from biological triplicates of either *dcl1*-*5* globular embryos or wild-type embryos from eight stages. As shown in the key, libraries are color-coded according to their stage: pg = preglobular; gl (WT) = wild-type globular; gl (*dcl1*-*5*) = *dcl1*-*5* globular; eh = early heart; lh = late heart; et = early torpedo; lt = late torpedo; bc = bent cotyledon; mg = mature green. **(B)** Volcano plot of log_2_-transformed transcript levels in (*dcl1*-*5* TPM + 1)/(wild type TPM + 1) (*x* axis) and log_10_-transformed Benjamini–Hochberg-adjusted *P* values based on DESeq2 (Love et al., 2014) in *dcl1*-*5* as compared with wild-type (*y* axis; *bottom*). Red and blue indicate transcripts with *P* values < 0.01 and ≥2-fold increased levels in *dcl1*-*5* or wild-type embryos, respectively. Histogram of the total numbers of transcripts across different *dcl1*-*5*/wild-type transcript fold-changes are shown (*top*). The number of significantly decreased (sig. dec.) and increased (sig. inc.) transcripts are indicated. **(C)** Violin plots of log_2_-transformed transcript levels in (*dcl1*-*5* + 1)/(wild-type + 1) for embryo phase-enriched marker transcripts as defined in Hofmann et al. (2019). Transcripts with ≥1 TPM in either *dcl1*-*5* or wild-type globular embryos (All; *n* = 18,420) and enriched in either Pre-cotyledon (*n* = 107), Transition (*n* = 127), Mature green (*n* = 48), or Dry seed (*n* = 183) phases. *P* values < 0.01 and *P* values < 10^−6^ are indicated by ^*^ and ^***^, respectively (two-tailed K–S test). **(D)** Cumulative distributions of (*dcl1*-*5* TPM + 1)/(wild-type TPM + 1) transcript levels (log_2_) for all transcripts with ≥1 TPM in either *dcl1*-*5* or wild-type globular embryos (All; black), transcripts confidently predicted computationally (2 < Allen scores ≤ 4, dark purple; Allen scores ≤ 2, light purple), and high-confidence targets detected by the tool “EndCut” (nanoPARE, orange; ^***^P values = 3.96 × 10^−11^).

To assess how miRNAs influence embryonic transcript levels, we profiled transcriptomes from *dcl1*-*5* globular embryos in which miRNA levels and cleavage activities were highly reduced (Figures 2B, 3A, 3C, 4A, and 4B). Principal component analysis of *dcl1*-*5* and wild-type embryonic transcriptomes (Hofmann et al., 2019) revealed that *dcl1*-*5* biological triplicates clustered together in a group that was separate from the wild-type transcriptomes (Figure 5A). This suggested that the loss of miRNAs resulted in large-scale changes in transcript populations. Indeed, 3,321 and 1,951 genes had at least 2-fold significantly increased and decreased transcript levels in *dcl1*-*5* relative to wild-type globular embryos, respectively (DESeq2; Benjamini–Hochberg-adjusted *P* values < 0.01; Figure 5B; Supplemental Data Set 4). Considering that 18,420 genes had ≥1 transcripts per million (TPM) in either wild-type or *dcl1*-*5* globular embryos, this indicated that 28.6% of the transcriptome is significantly altered in *dcl1*-*5* embryos. Differences due to RNA contamination from nonembryonic seed tissues could be ruled out by applying the tissue-enrichment test (Schon and Nodine, 2017), which revealed no significant RNA contamination in either the wild-type or *dcl1*-*5* samples (Supplemental Figure 7). These large-scale transcriptome changes may be related to the precocious activation of embryo maturation gene expression programs as reported in Nodine and Bartel (2010) and Willmann et al. (2011). To test whether transcriptomes from *dcl1*-*5* globular embryos resembled those from later stages of development, we examined the levels of transcripts that were specifically enriched during one of four main phases of embryogenesis and seed development (Hofmann et al., 2019) in *dcl1*-*5* as compared with wild-type globular embryos. The levels of Transition, Mature green, and Dry seed phase marker transcripts were significantly increased in *dcl1*-*5* globular embryos (*P* values < 10^−6^, two-tailed K-S tests; Figure 5C). Therefore, miRNA-deficient *dcl1*-*5* embryos indeed prematurely activate late-stage gene expression programs.

Because we detected relatively few miRNA targets with nanoPARE as compared with the total number of differentially expressed genes (Figures 4B and 5B; Supplemental Data Set 4), we reasoned that either many miRNA targets were not detected with nanoPARE or that the large-scale changes in *dcl1*-*5* transcriptomes were mostly a consequence of miRNA target derepression. Plant miRNA targets can be predicted at various confidence levels depending on the frequency and position of the miRNA:target duplex mismatches (i.e. Allen scores; Allen et al., 2005). Whereas the levels of miRNA targets detected with nanoPARE were significantly increased in *dcl1*-*5* relative to wild-type embryos as compared with all expressed genes (2.8-fold; *P* value = 3.96 × 10^−11^, two-tailed K–S test), the levels of targets confidently predicted computationally (i.e. Allen scores ≤2 or ≤4), including bona fide postembryonic targets, were not substantially increased in *dcl1*-*5* (Figure 5D). These results are consistent with the nearly complete depletion of miRNAs in *dcl1*-*5* embryos resulting in the loss of cleavage and repression of dozens of targets and the consequential misregulated miRNA target activities having a large impact on embryonic gene expression programs.

**Figure 6.**
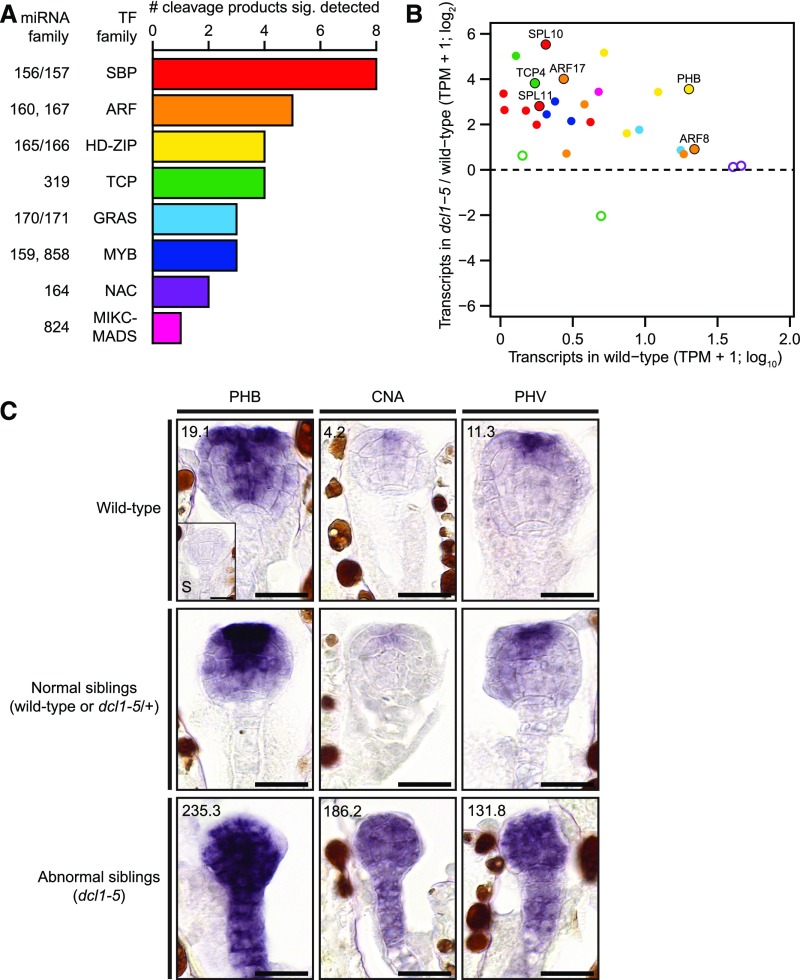
miRNA-Mediated Cleavage and Repression of Transcripts Encoding Transcription Factors **(A)** Stacked bar chart indicating the number of transcription factor family members for which high-confidence cleavage products were detected during embryogenesis. Transcription factor families, as well as the miRNA families that mediate their cleavage are labeled. **(B)** Scatterplots illustrating the levels of transcripts encoding transcription factors for which high-confidence cleavage products were detected according to their levels in wild type (TPM + 1; log_10_) and relative fold-changes in log_2_-transformed (*dcl1*-*5* TPM + 1)/(wild-type TPM + 1). Transcripts with >1 TPM in either wild-type or *dcl1*-*5* embryos are shown (*n* = 28). Significantly increased transcripts (*n* = 24; DESeq2 Benjamini–Hochberg-adjusted *P* values < 0.01; DESeq2) are indicated by points filled with colors representing various transcription factor families as shown in **(A)**. Six targets selected for further analyses are labeled directly above each corresponding point and are also indicated with outlines. **(C)** Representative images of RNA in situ hybridizations with probes antisense to either PHB (*left*), CNA (*middle*), or PHV (*right*) transcripts on sections of embryos. RNA in situs were performed on embryos from either self-pollinated wild-type (*top*) or *dcl1*-*5*/+ plants. Embryos from self-pollinated *dcl1*-*5*/+ plants were further classified into either normal (*middle*, wild-type or *dcl1*-*5*/+), or abnormal (*bottom*, *dcl1*-*5*) siblings based on morphology. A sense control for PHB (“S”) is displayed in the inset of the top left panel. Numbers in top left corners of wild-type and abnormal sibling images indicate transcript levels (TPMs) determined by mRNA-Seq. Scale bars = 20 µm.

Thirty of the 59 high-confidence miRNA targets detected with nanoPARE, encoded transcription factors belonging to eight different families, including those containing the ARF, GRAS, HD-ZIP, MADS-box, MYB, NAC, SBP, and TCP domains (Figure 6A). Twenty-eight of these had transcripts >1 TPM in either wild-type or *dcl1*-*5* globular embryos, and remarkably, 24 (85.7%) were significantly upregulated in *dcl1*-*5* as compared with wild-type embryos (Figure 6B). RNA in situ hybridizations of three miR165/166 target RNAs encoding class III HD-ZIP transcription factors (i.e. PHABULOSA [PHB], CORONA [CNA], and PHAVOLUTA [PHV]) in wild-type embryos were congruous with previous reports (McConnell et al., 2001; Prigge et al., 2005; Smith and Long, 2010) and transcriptome analyses (Hofmann et al., 2019). Consistent with their upregulation in *dcl1*-*5* embryos, the PHB, CNA, and PHV transcripts had increased signals throughout embryos, including ectopic localization in the basal and peripheral regions of *dcl1*-*5* embryos (Figure 6C). Together with the observation that miR165/166 and its target transcripts had opposite localization patterns in heart-staged wild-type embryos (Figures 3C and 6C), the ectopic localization of class III HD-ZIP transcripts further supports the notion that miR165/166 helps define the proper localization patterns of their target transcription factors.

### miRNA-Directed Repression Across Embryonic Cell-Types

Embryonic miRNAs direct the cleavage and repression of at least 30 transcripts that encode transcription factors (Figures 4B, 6A, and 6B). To examine miRNA-mediated gene repression at cellular resolution, we employed a GFP-based miRNA sensor system (Nodine and Bartel, 2010). Either a random 21-nucleotide non-genome–matching sequence (i.e. scrambled sensor) or 20- to 22-nucleotide embryonic miRNA target sites detected by nanoPARE for miR156/157 (SPL10/11), miR160 (AUXIN RESPONSE FACTOR 17, ARF17), miR165/166 (PHB), miR167 (ARF8), or miR319 (TCP4) were included in nuclear-localized GFP constructs under the control of a ubiquitous promoter. If the miRNA mediates repression, then the sensor transgene containing the corresponding target site was expected to produce less GFP signal.

**Figure 7.**
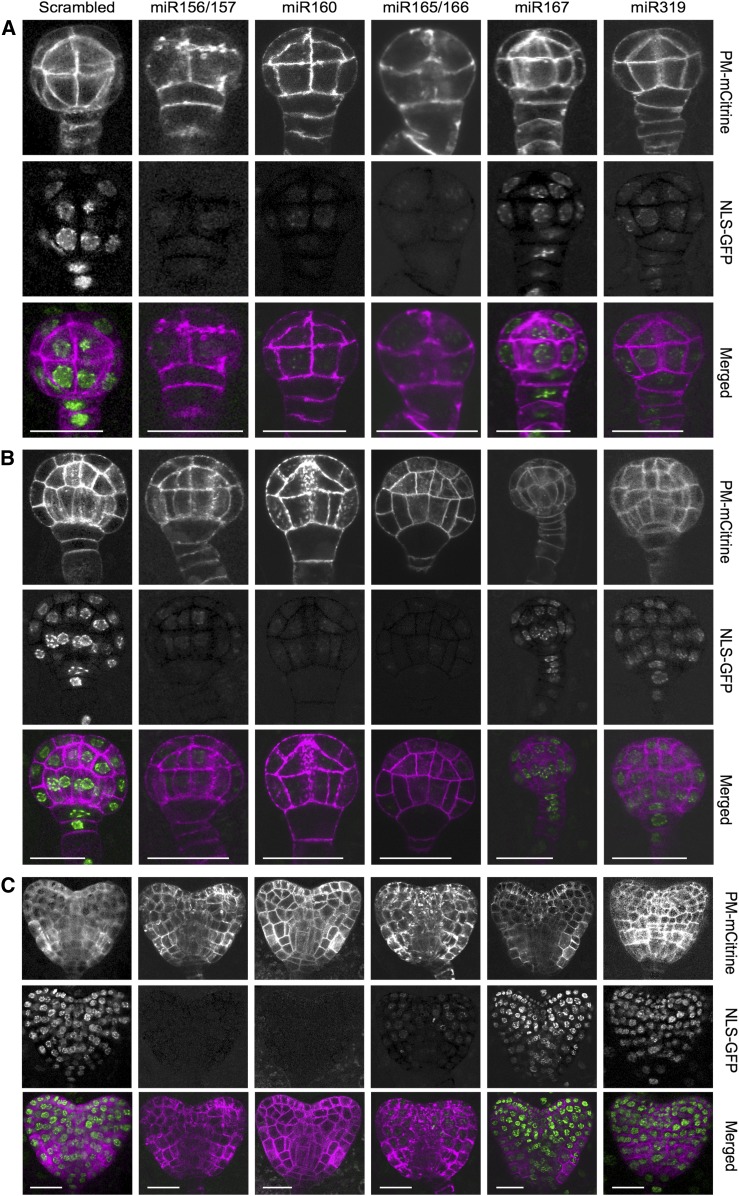
miRNA-Directed Repression Across Embryonic Cell Types Representative confocal microscopy images of preglobular **(A)**, globular **(B)** and early heart **(C)** staged Col-0 embryos expressing a plasma membrane-localized mCitrine fluorescent protein under the control of the embryo-specific *WOX2* promoter (pWOX2:mCitrine-SYP122) and ubiquitously expressing transcripts encoding nuclear-localized HTA6-GFP (pUBI3:HTA6-GFP) with 20- to-22-nucleotide sequences that are either a non-genome–matching random sequence (scrambled) or target sites detected in embryos with nanoPARE for SPL10/11 (miR156/157), ARF17 (miR160), PHB (miR165/166), ARF8 (miR167), or TCP4 (miR319), as labeled at the top. Images with signal corresponding to plasma-membrane localized pWOX2:mCitrine-SYP122 (PM-mCitrine) and pUBI3:HTA6-GFP (NLS-GFP) are indicated, as well as images from the merged channels color-coded in magenta and green, respectively. Scale bars = 20 μm. Brightness and contrast were uniformly adjusted for each image using the “Best Fit” tool from the ZEN software (Zeiss; see Methods).

As expected based on our sRNA-seq, nanoPARE, and *dcl1*-*5* mRNA-seq data sets, all five miRNA sensors had reduced GFP signal as compared with scrambled sensors in at least one early embryonic stage (Figure 7). Sensors for miR156/157 and miR165/166 had strongly decreased levels throughout preglobular and globular embryos. At the heart stage, miR156/157 sensors were repressed throughout embryos, and miR165/166 sensors had increased levels in apical regions. miR156/157 and miR165/166 sensor activities were generally consistent with the results of RNA in situ analyses (Figures 3C and 6C) and previous reports (Nodine and Bartel, 2010; Smith and Long, 2010; Miyashima et al., 2013). However, the miR165/166 sensors used in our study were repressed in more cell types, likely due to the use of different sensor constructs. The observed increases in miR165/166 target transcript levels throughout *dcl1*-*5* embryos further supports the idea that miR165/166 has broad repressive domains in early embryos (Figure 6C).

The levels of miR160 sensors were also reduced throughout heart stage embryos (Figure 7). Because miR160 was detectable only in the provasculature by in situ hybridizations on sections of heart stage embryos (Figure 3C), the sensor approach appears to have better sensitivity than in situ hybridization. In fact, miR160 was detected throughout late-staged embryos when performing more sensitive whole-mount in situs (Ghosh Dastidar et al., 2016). miR167 and miR319 sensors were weakly repressed in preglobular embryos and exhibited dynamic patterns thereafter (Figure 7). At the globular stage, miR167 sensor signals were reduced at the base of the suspensor and progressively decreased acropetally. By the heart stage, miR167 sensors had reduced signals throughout the suspensor and base of the embryo proper, as well as in the shoot meristem precursors. miR319 sensors were weakly repressed throughout globular-staged embryos, and exhibited stronger repression in the basal regions of heart-staged embryos. Altogether, the sensor dynamics support the nanoPARE and *dcl1*-*5* mRNA-seq data sets, and indicate that miRNAs can differentially mediate the cleavage and repression of targets, including those encoding transcription factors, across early embryonic cell types and developmental stages.

### miRNA-Mediated Repression of Transcription Factors is Required for Embryo Morphogenesis

**Figure 8.**
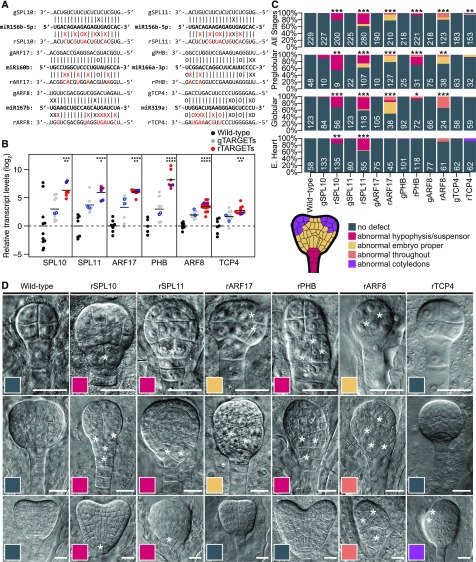
miRNA-Mediated Repression of Transcription Factors is Required for Embryo Morphogenesis **(A)** Schematics of six miRNA target sites in transcripts encoding transcription factors selected for mutagenesis. The dominant miRNA isoforms in globular stage embryos for each family are shown. Basepairing interactions with either wild-type target sites (genomic, gTARGET) or miRNA-resistant target sites (resistant, rTARGET) are indicated above and below, respectively. Mutations introduced by site-directed mutagenesis are labeled in red. Watson-Crick base-pairing (I), non-basepairing (X), and G:U wobbles (O) for each pair are indicated. **(B)** miRNA target transcript levels in flowers from wild-type plants, or plants expressing either wild-type (gTARGET) or miRNA-resistant (rTARGET) versions of the target transcripts. qRT-PCR values were internally normalized with transcript levels from the *eIF4A1* housekeeping gene and then divided by the levels observed in wild-type plants and log_2_-transformed. Each dot represents the mean of two technical replicates from an independent transgenic line (first generation; T_1_ lines) and is color-coded according to the key. Horizontal bars represent means ± se between all lines. Asterisks indicate whether the transcript levels observed in rTARGET lines were significantly different as compared with either wild type (*top*) or gTARGET controls (*bottom*; two-tailed Student’s *t* tests; ^****^, ^***^, ^**^, and ^*^ represent *P* values < 0.0001, < 0.001, < 0.01, and < 0.05, respectively). Points corresponding to lines selected for phenotypic analyses are outlined in blue. **(C)** Stacked bar plot illustrating the proportions of phenotypes observed for embryos from self-pollinated wild-type plants, or T_1_ plants expressing either wild-type (gTARGET) or miRNA-resistant (rTARGET) versions of target transcripts in all stages examined, or in preglobular, globular, or early heart (“E. Heart”) stages as indicated. Genotypes are labeled at the bottom, numbers at the base of each bar indicate the number of embryos examined, and phenotypes are color-coded according to the key. Asterisks indicate whether the number of abnormal embryos was significantly greater than for wild-type (one-tailed Fisher’s exact test; ^***^, ^**^, and ^*^ represent *P* values < 0.0001, < 0.001, and < 0.01, respectively). **(D)** Representative Nomarski images of embryos from wild-type plants, or expressing miRNA-resistant targets at the preglobular (*top*), globular (*middle*), or early heart (*bottom*) stages. Genotypes are labeled above, and asterisks indicate abnormal cell divisions. Individual panels are color-coded according to the key in **(C)**. Scale bars = 20 μm. Brightness and contrast were adjusted with the software “Photoshop” (Adobe) uniformly for each image to improve visibility (see Methods).

The often dynamic repressive activities directed by miRNAs help define the spatiotemporal domains of transcription factors and likely have a large impact on embryonic transcriptional regulatory networks, including those that help define the future plant body plan (Figures 4 to 7; Nodine and Bartel, 2010; Seefried et al., 2014). To determine how miRNA-mediated cleavage of transcripts encoding transcription factors contributes to embryo morphogenesis, we selected six miRNA:target interactions to investigate further, including miR156/157:SPL10, miR156/157:SPL11, miR160: ARF17, miR165/166:PHB, miR167:ARF8, and miR319:TCP4 (Figures 6B and 8A).

To generate target transgenes resistant to miRNA-mediated cleavage (i.e. resistant targets), we cloned these six target loci, including upstream and downstream intergenic sequences with endogenous cis-regulatory elements (4.9–7.8 kb depending on the locus), and introduced two to seven synonymous mutations into the corresponding miRNA target sites using site-directed mutagenesis to abolish miRNA binding (Figure 8A). Because phenotypes resulting from miRNA-resistant transgenes should be interpreted with caution (Li and Millar, 2013), we performed the following experiments to control for transgene-induced artifacts: To control for potential effects unrelated to the disruption of the miRNA binding sites, we generated constructs for each resistant target whereby the corresponding miRNA binding sites were left intact (i.e. genomic targets; Figure 8A). We transformed the resistant and genomic target constructs into wild-type plants and selected 6–17 independent transgenic lines for each construct. The postembryonic phenotypes of resistant target lines were consistent with previous reports (Mallory et al., 2005; Wu et al., 2006, 2009; Supplemental Figures 8A and 8B). To select representative lines for miRNA-resistant targets and their controls for further characterization, we performed quantitative RT-PCR (qRT-PCR) on target transcripts in floral bud total RNA from wild type or their respective genomic or resistant target lines (113 total independent lines; Figure 8B). Significantly higher transcript levels were observed in all sets of resistant lines relative to wild type and corresponding genomic target control lines (*P* values < 0.001 and 0.05, respectively; *t* tests; Figure 8B). Based on the qRT-PCR analyses, we selected at least two representative lines for each construct to examine for embryo abnormalities (26 total independent lines plus wild type; Figure 8B). The selection of multiple representative miRNA-resistant and control lines based on qRT-PCR together with miRNA-resistant lines phenocopying previously reported postembryonic phenotypes upon miRNA knock-down strongly suggests that the phenotypes exhibited by the miRNA-resistant target lines used in this study are due to abolishing miRNA target sites rather than transgene-induced artifacts. Indeed, although we observed a modest increase in target transcript levels in control lines based on qRT-PCR, only lines with mutations in miRNA binding sites exhibited significantly increased numbers of abnormal phenotypes (Figure 8C; Supplemental Figure 8C).

We phenotyped 2,682 preglobular to heart stage embryos from the 26 representative independent transgenic lines and wild type. Distinct morphological defects were observed for each miRNA-resistant target (Figure 8C), and these were reproducible among independent transgenic lines (Supplemental Figure 8C). Consistent with our previous report, miR156/157-resistant SPL10 and SPL11 transgenic embryos exhibited abnormal divisions in their uppermost suspensor and hypophysis cells during early embryogenesis and failed to generate lens-shaped cells (i.e. precursors to the root quiescent center; Figure 8D). Embryos with miR160-resistant ARF17 constructs had abnormal divisions in the embryo proper beginning at the preglobular stage when, for example, the protoderm layer failed to form on one side of the embryo (Figure 8D). Later during the globular stage, embryos with miR160-resistant ARF17 transgenes had abnormal divisions in the basal region of the subprotoderm. Preglobular and globular embryos with miR165/166-resistant PHB transgenes had abnormal divisions in the hypophysis (Figure 8D). Embryos from self-pollinated miR160-resistant ARF17 and miR165/166-resistant PHB lines appeared normal at the heart stage, but this may be due to lethality or developmental delay of embryos expressing the miRNA-resistant transgenes. Preglobular embryos with miR167-resistant ARF8 transgenes had defective divisions in the embryo proper, and then throughout globular and heart embryos (Figure 8D). Embryos with miR319-resistant TCP4 transgenes were morphologically normal during the preglobular and globular stages, but at the heart stage had a low, but significantly increased, number of embryos with defective cotyledon outgrowth (Figure 8D).

Because most of the miRNA-resistant target lines were sterile and had abnormal flower morphologies (Supplemental Figure 8B), we crossed wild-type plants (as the female parent) with plants that were either wild-type or transgenic for miRNA-resistant constructs to determine whether the phenotypes observed from self-pollinated miRNA-resistant target lines were due to zygotic or maternal sporophytic effects of the transgenes. All crosses between wild-type maternal plants and resistant target lines yielded significantly more abnormal embryos than wild-type crosses (*P* values < 0.01; Fisher’s exact test), and the progeny exhibited phenotypes similar to the self-pollinated resistant targets (Supplemental Figures 8D and 8E). Therefore, specific interactions between miRNAs and transcripts encoding transcription factors are morphologically required in a variety of embryonic cell types.

## DISCUSSION

We developed a sRNA-seq library preparation protocol that highly enriches for sRNAs, as well as reproducibly and accurately measures their levels from low amounts of total RNA (i.e. ≥1 ng). We expect this method to be useful for profiling sRNA populations from difficult-to-obtain samples, including plant and animal reproductive tissues. Here, we used this low-input sRNA-seq method to profile sRNA populations across Arabidopsis embryogenesis. The sRNA-seq and nanoPARE data sets reported in this study, as well as the transcriptome data sets produced from the same stages (Hofmann et al., 2019), provide a solid foundation for the characterization of noncoding and coding RNA populations in plant embryos. For example, miRNAs comprise only a fraction of the embryonic sRNA population, and these integrated sRNA-seq, nanoPARE, and mRNA-seq data sets will also enable the systematic characterization of additional sRNAs present in early embryos, including small interfering RNAs involved in the establishment of epigenetic marks and associated transcriptional gene silencing events.

In this study, we generated a catalog of 354 miRNAs present during embryogenesis and applied our recently developed nanoPARE method to identify 59 high-confidence embryonic miRNA targets. We found high-confidence miRNA-directed cleavage products for only 22 out of 115 detected embryonic miRNAs, suggesting that many miRNAs may not be directing target cleavage in the stages and conditions examined. Although this could be partially explained by limited sensitivity of the nanoPARE method, our observation that targets detected by nanoPARE, but not those only confidently predicted computationally, had globally increased transcript levels in *dcl1*-*5* embryos suggests that we have identified the majority of cleavage events. Moreover, we detected miRNA-directed cleavage products of all targets with published evidence supporting their existence during Arabidopsis embryogenesis (Palatnik et al., 2003; Mallory et al., 2005; Nodine and Bartel, 2010; Smith and Long, 2010; Knauer et al., 2013; Miyashima et al., 2013; Takanashi et al., 2018). We propose that many of the detected miRNAs function as fail-safes to prevent the aberrant accumulation of target transcripts or have already executed their functions during earlier stages of development. For instance, we were unable to detect targets for any of the five miRNA families that were abundant and enriched at the earliest stages of embryogenesis. The levels of these miRNAs decreased dramatically during early embryogenesis, and they may function directly after fertilization and before the earliest stage profiled with nanoPARE (i.e. preglobular stages comprised of 8-cell and 16-cell staged embryos).

As a proof-of-principle of this resource’s utility, we focused on the miRNA-mediated regulation of transcription factors in this study. We and others have demonstrated that miRNAs are required for pattern formation and proper developmental timing of gene expression programs during Arabidopsis embryogenesis (Nodine and Bartel, 2010; Willmann et al., 2011; Seefried et al., 2014). Indeed, the more comprehensive *dcl1*-*5* embryo transcriptome data set and analyses presented here further supports the concept that miRNAs have a large impact on the embryonic transcriptome, including the prevention of precocious expression of genes characteristic of the maturation phase of embryogenesis and related to oil body biogenesis, lipid storage, and other seed maturation processes (Hofmann et al., 2019). Because <5% of the transcripts whose levels significantly increased in *dcl1*-*5* embryos were directly cleaved and repressed by miRNAs, the vast majority of misregulated genes in *dcl1*-*5* embryos are likely downstream of miRNA targets. Interestingly, 30 of the 59 embryonic miRNA high-confidence targets identified encoded transcription factors. Their derepression in miRNA-deficient *dcl1*-*5* embryos, along with associated misregulated downstream transcriptional cascades, may largely explain why thousands of transcripts are present at different levels in *dcl1*-*5* as compared with wild-type embryos.

Together with previous studies, our results indicate that multiple miRNAs are required for embryo morphogenesis and pattern formation. We previously demonstrated that miR156/157 prevents the accumulation of SPL transcription factors and the resulting expression of maturation phase genes during early embryogenesis (Nodine and Bartel, 2010). Although decreased SPL10/11 levels could suppress miRNA-deficient *dcl1*-*5* phenotypes, abolishing miR156/157:SPL10/11 interactions was not sufficient to fully phenocopy *dcl1*-*5* embryos. This suggested that additional miRNA:target interactions are required for embryonic pattern formation. Accordingly, the hypophysis and suspensor division defects observed in embryos expressing miR156/157-resistant SPL10/11 and miR165/166-resistant PHB, as well as the embryo proper defects of miR160-resistant ARF17 embryos, were also observed in *dcl1*-*5* embryos (Figure 8D; Nodine and Bartel, 2010). Moreover, the pleiotropic defects exhibited by miR167-resistant ARF8 embryos and cotyledon initiation defects observed in miR319-resistant TCP4 embryos further support the idea that multiple miRNAs are required for proper embryo morphogenesis. Interestingly, preglobular stage miR160-resistant ARF17, miR165/166-resistant PHB, and miR167-resistant ARF8 embryos often exhibit more severe defects than we observed in *dcl1*-*5* (Figure 8D; Nodine and Bartel, 2010). Because homozygous *dcl1*-*5* embryos are lethal, they are derived from *dcl1*-*5*/+ parents. Therefore, it is possible that the *DCL1* gene products inherited from diploid sporocytes are sufficient to produce miRNAs in gametophytes or early embryos, as previously proposed for other essential genes (Muralla et al., 2011). Additionally, redundant activities of other *DCL* genes may partially compensate for the loss of *DCL1* in preglobular embryos. Consistent with both of these explanations, miR165/166 and miR167 levels were highly reduced, but not eliminated, in globular stage *dcl1*-*5* embryos (Supplemental Figure 3; Supplemental Data Set 2).

The developmental progression of miRNA-resistant target phenotypes generally corresponds well with the spatiotemporal dynamics of the corresponding miRNAs and their activities. miRNA-resistant transgenes generally caused phenotypes in the same cell-types in which the corresponding miRNAs were active (Figures 7 and 8D). One exception was the defective cotyledons observed in miR319-resistant TCP4 embryos. Although cotyledon initiation occurs at the heart stage when miR319 activities were increased (Figure 4B), and the cotyledon defects were in agreement with previously reported seedling phenotypes (Palatnik et al., 2003), miR319 was more active in basal regions of embryos (Figure 7). Therefore, gene-regulatory processes downstream of miR319-mediated repression of TCP4 may be non-cell–autonomously required for cotyledon formation. By contrast, miR160-resistant ARF17 and miR165/166-resistant PHB exhibited abnormal phenotypes in the cell types in which their highest levels or repressive activities were detected. For example, miR160-resistant ARF17 had defects in the subprotoderm of the embryo proper, which is congruent with higher miR160 levels in these cell types. Together with previously reported phenotypes of embryos with *mir160a* loss-of-function mutations (Liu et al., 2010), our results indicate that miR160-mediated repression of the ARF17 transcription factor is required for proper subprotodermal cell division patterns.

The observation that miR165/166-mediated repression of target HD-ZIP transcripts occurs in basal embryonic regions indicates that miR165/166 helps define HD-ZIP transcription factor localization domains in early embryos (Figures 4B, 6, and 7; McConnell et al., 2001; Smith and Long, 2010; Miyashima et al., 2013;). Accordingly, embryos expressing miR165/166-resistant PHB exhibited abnormal divisions typically in basal regions of the embryo (Figures 8C and 8D), indicating that miR165/166:PHB interactions are required in these cell types for proper morphogenesis. miR167 and its repression of ARF8 are required in the maternal sporophytic tissues for proper embryogenesis (Wu et al., 2006; Yao et al., 2019). Interestingly, plants containing miR167-resistant ARF8 transgenes had similar phenotypes when crossed as the pollen donors to wild-type maternal lines. This indicates that miR167-mediated repression of ARF8 is required in embryos for proper morphogenesis, which is similar to the five other miRNA:target interactions we characterized (Supplemental Figures 8D and 8E). Altogether, these data support a model whereby the posttranscriptional regulation of transcription factor gene-regulatory networks by several miRNAs is critically important for the establishment of the plant body plan during early embryogenesis. The resources and phenotypes described in this study provide multiple entry points to further characterize how the miRNA-mediated repression of transcripts, including those encoding transcription factors, contributes to the initial cellular differentiation events that operate at the beginning of plant life.

## METHODS

### Plant Material, Growth Conditions, and RNA Isolation

The *dcl1*-*5* (McElver et al., 2001) and *xrn4*-*5* (Souret et al., 2004) alleles in the Arabidopsis (*Arabidopsis thaliana*) Col-0 accession background, together with Col-0, were grown in a climate-controlled growth chamber at 20°C to 22°C under a 16-h light/8-h dark cycle. Plants were grown under incandescent lights at 130 to 150 μmol/m^2^/s. Embryos were dissected and total RNA was extracted at a similar time of day (1:00pm–5:00pm) as described in Hofmann et al. (2019). Except for the bent-cotyledon stage samples, all other total RNA samples pooled from 50 Col-0 embryos were used to generate mRNA-seq data sets (Hofmann et al., 2019) and the sRNA-seq and nanoPARE data sets reported in this study. Total RNA from 7-day–old *xrn4*-*5* seedling roots and shoots grown vertically on 0.5× Murashige and Skoog plates were isolated as described in Schon et al. (2018). In this study, biological replicates of mRNA-seq, nanoPARE, and mRNA-seq data sets were from pools of RNA collected from different embryos, leaves, flowers, roots, or shoots on different days.

### Low-Input sRNA-Seq

A quantity of 18-nucleotide to 30-nucleotide RNAs were purified from ≥80% (v/v) of the total RNA from each sample (from 50 pooled embryos) using denaturing polyacrylamide-urea gels as described previously by Grimson et al. (2008). Size-selected sRNAs were precipitated overnight at −20°C with 2.5× volumes of ice-cold 100% ethanol and 1 μL of GlycoBlue (Thermo Fisher Scientific) and resuspended in 7.5 μL of nuclease-free water. This sample was used as input for the NEBNext Multiplex Small RNA Library Prep Set for Illumina kit (cat. no E7300; New England Biolabs) according to the manufacturer’s recommendations with the following modifications: Adapters used for 3′ and 5′ ligations to sRNAs, and SR RT primers to generate sRNA cDNAs, were diluted to 25% (v/v) of the amounts recommended for ≥500 ng of total RNA. Various numbers of PCR cycles were used to amplify cDNAs: 14, 16, 18, and 20 PCR cycles for early heart and later staged samples, and 18, 20, 22, and 24 PCR cycles for globular and earlier staged samples. Final amplicons were run on a 90% (v/v) formamide/8% (w/v) acrylamide gel at 5W for ∼30 min, followed by 30W for ≥2 h, and stained with SYBR Gold (1:10,000; Thermo Fisher Scientific). Fluorescence intensities of amplicons were examined across the PCR cycles, 137-bp to 149-bp products (corresponding to 18- nucleotide to 30- nucleotide sRNAs) with nonsaturated signals were gel-purified, and after DNA precipitation, pellets were resuspended in 15 μL of Elution Buffer (Qiagen). To control for library quality, sRNA-seq libraries were examined on a DNA HS Bioanalyzer Chip (Agilent Technologies), and those with the expected size range were sequenced on a HiSeq 2500 instrument (Illumina) in 50 base single-end mode (Supplemental Data Set 1).

The tool “Cutadapt” (Martin, 2011) was used to trim adapter sequences from sRNA-seq reads, and 18–30-base sequences that contained an adapter were retained. The trimmed sequences were aligned to The Arabidopsis Information Resource 10 (TAIR10) genome (Lamesch et al., 2012) with the tool “STAR” (Dobin et al., 2013) requiring no mismatches and allowing ≤100 multiple end-to-end alignments. The resulting “SAM” files were processed with the “readmapIO.py” script to reassign multimappers with a “rich-get-richer” algorithm as described in Schon et al. (2018). “Output bedFiles” were sorted, condensed, and normalized for total genome-matching reads. The “map” function in the software “BEDtools” (Quinlan and Hall, 2010) was used to quantify the number of reads mapping to the same strand and overlapping ≥80% of mature miRNAs as annotated in TAIR10 and “miRBase21” (Kozomara et al., 2019). Statistical analyses and associated figures were generated with the statistical computing package “R” (R Core Team, 2018).

### nanoPARE and mRNA-Seq

For transcriptome analyses, “Smart-seq2” libraries (Picelli et al., 2013) were generated from *dcl1*-*5* embryos selected from self-fertilized *dcl1*-*5*/+ plants based on their abnormal morphologies as previously described by Hofmann et al. (2019). These were sequenced on a HiSeq 2500 instrument (Illumina) in 50 basepaired-end mode (Supplemental Data Set 1). Transcriptome analyses were performed as described by Hofmann et al. (2019), except that TAIR10 transposable element gene models were also included in the “Kallisto”-based pseudo-alignments (Bray et al., 2016). The program “DESeq2” (Love et al., 2014) with default settings was used to compute *P* values for the wild-type and *dcl1*-*5* transcriptome comparisons.

The nanoPARE libraries presented in this study were generated as previously described by Schon et al. (2018) with the following exceptions: For all embryonic samples other than those from the bent-cotyledon stage, the same cDNA pools used in our previous transcriptome analysis (Hofmann et al., 2019) were also used as input for nanoPARE library preparation. The nanoPARE libraries were sequenced on a Hi-Seq 2500 instrument (Illumina) in 50 base single-end mode (Supplemental Data Set 1). Analysis of the nanoPARE data was performed as described by Schon et al. (2018), except that all capped features identified in the embryonic series were merged with those from published floral bud samples (Schon et al., 2018) and used to mask capped features from the transcript-level “bedGraph” files. Additionally, we used the tool “EndCut” (Schon et al., 2018) to test for significant target sites for 164 miRNAs detected ≥1 RPM in at least one embryonic stage. nanoPARE libraries from all postembryonic tissues were analyzed in an identical manner.

### RNA In Situ Hybridizations

miRNA in situs on embryo sections were performed based on a whole-mount in situ hybridization method (Ghosh Dastidar et al., 2016). Sample preparation leading up to probe hybridization was performed as described by Nodine et al. (2007), except that a LOGOS Microwave Hybrid Tissue Processor (Milestone Medical) was used for tissue embedding, and the samples were fixed with a solution of 0.16 M n-[3-Dimethylaminopropyl]-n′-ethylcarbodiimide hydrochloride in Methylimidazole-NaCl after the proteinase K digestion step as follows: First, slides with adhered embryo sections were transferred to 1× PBS and washed 2×, and then incubated in a staining dish containing freshly prepared methylimidazole-NaCl for 10 min at room temperature (2×). The slides were then transferred to 0.16 M n-[3-Dimethylaminopropyl]-n′-ethylcarbodiimide hydrochloride in Methylimidazole-NaC solution and incubated for 2 h at 60°C, and subsequently washed 2× in 1× PBS for 5 min each before probe hybridization. Dual DIG-labeled LNA-modified oligos antisense to miR124, miR156a-f, miR159a, miR160a-c, or miR166a-f isoforms were used at a final concentration of 20 nM (Supplemental Table), and the rest of the probe hybridization procedure, as well as subsequent washing, antibody, and colorimetric reactions were as described by Nodine et al. (2007). Slides were imaged on an automated Pannoramic SCAN 150 Slide Scanner (3DHISTECH) and collected with the associated “Pannoramic Viewer” software. Images of ≥50 embryos from >5 independent sets of experiments were recorded.

The mRNA in situs were performed as previously described by Nodine et al. (2007). Probes antisense to CNA, PHB, and PHV were generated from cDNAs by introducing T7 promoters via PCR as described previously by Hejátko et al. (2006; Supplemental Table).

### Generation of Transgenic Lines

Nuclear-localized GFP-based sensor constructs with miR156/157:SPL10/11 target sites (GTG​CTC​TCT​CTC​TTC​TGT​CA) in the 5′ UTR and under the control of the potato (*Solanum tuberosum*) *UBI3* promoter were generated as previously described by Nodine and Bartel (2010). A similar strategy was also employed to create constructs with the miR160:ARF17 (TGG​CAT​GCA​GGG​AGC​CAG​GCA), miR165/166:PHB (TGG​GAT​GAA​GCC​TGG​TCC​GG), miR167:ARF8 (TTA​GAT​CAG​GCT​GGC​AGC​TTG​T), and miR319:TCP4 (AGA​GGG​GTC​CCC​TTC​AGT​CCA​G) target sites detected in embryos with nanoPARE. As a negative control, we also generated identical constructs except with a random 21-nucleotide sequence (CCC​CGT​CTC​GCG​TCT​CAC​GCA) that does not map to the Arabidopsis genome. Constructs were transformed into Col-0 plants harboring nonsegregating transgenes for the mCitrine fluorescent protein fused to plasma membrane-localized SYP122 protein under the control of an embryo-specific *WOX2* promoter (Breuninger et al., 2008; pWOX2:mCitrine-SYP122) using the Agrobacterium floral dip method (Clough and Bent, 1998). At least two sensor lines were examined in the T_1_ and T_2_ generations.

Control genomic and miRNA-resistant SPL10 and SPL11 constructs were generated as described in Nodine and Bartel (2010). For control genomic ARF17 (gARF17), PHB (gPHB), and TCP4 (gTCP4) transgenic constructs, target loci including upstream and downstream intergenic sequences were PCR-amplified from Col-0 genomic DNA with primers containing overhangs for subsequent Gibson assembly. miR160-resistant ARF17 (rARF17), miR165/166-resistant PHB (rPHB), and miR319-resistant TCP4 (rTCP4) constructs were amplified as two separate fragments with overlaps to introduce specific mutations in the corresponding miRNA target sites. The backbones of the MultiSite-Gateway destination vectors pAlligatorG43 and pAlligatorR43 (Kawashima et al., 2014) were amplified for subsequent Gibson assembly, and genomic and resistant ARF17, PHB, and TCP4 plant transformation constructs were generated by Gibson Assembly (New England Biolabs) using the pAlligatorG43/R43 backbone and the target PCR fragments. For the control genomic ARF8 transgenic construct (gARF8), the ARF8 locus including upstream and downstream intergenic sequences was PCR-amplified from Col-0 genomic DNA and cloned into the pENTR/D-TOPO Gateway vector (Thermo Fisher Scientific). The miR167-resistant ARF8 construct (rARF8) was generated by PCR site-directed mutagenesis (New England Biolabs) of the gARF8 entry clone. Final plant transformation constructs were generated by Gateway LR reactions (Thermo Fisher Scientific) with pENTR-gARF8 or pENTR-rARF8, pDONR-L4R1-empty, and pDONR-R2L3-empty, and the Gateway destination vector pAlligatorR43 (red fluorescent protein). All primers are listed in Supplemental Table. The constructs were transformed into Col-0 using the Agrobacterium floral dip method (Clough and Bent, 1998), and transformants were selected based on GFP or red fluorescent protein selection marker fluorescence from pAlligatorG43/R43 (Bensmihen et al., 2004; Kawashima et al., 2014).

### qRT-PCR Analysis

Two clusters of floral buds were pooled from 8-week–old plants, snap-frozen in liquid nitrogen, homogenized using a Mixer Mill MM 400 (Retsch) for 30 s with maximum amplitude, and resuspended in 200 μL of TRIzol (Life Technologies). Total RNA was extracted using a Direct-zol RNA Kit (Zymo Research) according to the manufacturer’s instructions, and *DNa*seI treatment was performed on-column. Total RNA quality and quantity were determined with an Agilent Fragment Analyzer (AATI) using a standard RNA sensitivity kit (DNF-471). Two-hundred nanograms of total RNA samples with RNA Quality Number values >6.0 were used for cDNA synthesis together with the Oligo d(T)_18_ mRNA Primer (New England Biolabs) and SuperScript III Reverse Transcriptase (Thermo Fisher Scientific). The cDNA was diluted 10× with nuclease-free water, and 2 μL was used as a template for the qRT-PCR. qRT-PCR was performed on a LightCycler 96 Instrument (Roche) using gene-specific and control *elF4A* primers (Supplemental Table), and Fast SYBR Green Master Mix (Roche). Cycle threshold values were obtained using LightCycler 96 software (Roche), and relative quantification of transcripts (ΔΔCycle threshold values) was performed with an in-house “R” script. For each genotype, 6 to 17 individual first-generation transgenic (T_1_) lines were analyzed in technical duplicates.

### Microscopy

Self-pollinated siliques from at least two representative and independently generated first-generation transgenic (T_1_) lines for each miRNA-resistant and control constructs were harvested, and ovules were fixed and cleared in a solution composed of 8 g of chloral hydrate, 1 mL of water, and 1 mL of glycerol as described in Ohad et al. (1996). At least two representative T_2_ lines were also crossed as pollen donors to emasculated Col-0 flowers, and siliques were harvested 120 h after pollination. Embryos were examined with Nomarski optics on an Axio Observer Z1 with a CMOS camera (Zeiss). Images were acquired using the software “ZEN” (blue edition; Zeiss) imaging software and analyzed using the processing software “ImageJ/Fiji” (National Institutes of Health). To minimize potential bias, Nomarski images were examined by a person that did not acquire the images, and phenotypes were recorded before revealing sample identities. At least 38 embryos from 26 independent transgenic lines were examined for each construct (2,682 total embryos).

For confocal microscopy, whole seeds containing preglobular, globular, or early heart staged embryos were harvested, mounted in VectaShield Antifade Mounting Medium (Vector Laboratories), and imaged directly on a model no. LSM 780 Axio Observer (Zeiss) using a 488-nm excitation wavelength for both GFP and mCitrine; images were acquired using the same settings. The images were recorded and analyzed with the imaging software “ZEN” (black edition; Zeiss): Spectral unmixing was performed to differentiate between emission spectra of GFP (∼450 nm to 500 nm) and mCitrine (∼550 nm to 600 nm), and contrast and brightness were uniformly adjusted using the “Best Fit” tool of the ZEN imaging software. All images were cropped and rotated in the software “Photoshop” (Adobe). To increase the resolution and uniformity of the image panels, Nomarski images were further processed in Photoshop by applying the following tools to the whole image: “Image/Adjustments/Levels/Midtones Brighter” adjustments, “Auto Contrast” adjustments, and the “Unsharp Mask” filter.

### Accession Numbers, Data Acquisition, and Code Availability

All sequencing data generated in this study are available at the National Center for Biotechnology Information Gene Expression Omnibus (https://www.ncbi.nlm.nih.gov/geo/) under accession number GSE132066. Publicly available next-generation sequencing data were downloaded from the National Center for Biotechnology Information Gene Expression Omnibus (https://www.ncbi.nlm.nih.gov/geo/) with the following accession numbers: Col-0 sRNA-seq (GSE79414 and GSE98553), Col-0 mRNA-seq (GSE121236), and nanoPARE (accession number GSE112869). Custom software used to align sRNA-seq data to annotated mature miRNAs is available on GitHub (https://github.com/Gregor-Mendel-Institute/Plotnikova.2019).

### Supplemental Data

**Supplemental Figure 1.** Establishment of low-input sRNA-seq method
**Supplemental Figure 2.** Principal component analysis of embryonic and postembryonic miRNA populations
**Supplemental Figure 3.** Heat map with miRNA levels normalized by RPMs
**Supplemental Figure 4.** Embryo-enriched miRNAs
**Supplemental Figure 5.** mRNA 5′ ends of miRNA targets in *dcl1*-*5* mutant embryos
**Supplemental Figure 6.** miRNA and miRNA-mediated cleavage product correlations
**Supplemental Figure 7.** Tissue-enrichment test of wild-type and *dcl1*-*5* mutant embryo transcriptomes
**Supplemental Figure 8.** Control experiments for analysis of miRNA-resistant targets
**Supplemental Table.** Oligonucleotides used in this study
**Supplemental Data Set 1.** Summary of high-throughput data sets generated in or reanalyzed for this study
**Supplemental Data Set 2.** Levels of miRNAs detected during embryogenesis
**Supplemental Data Set 3.** Predicted miRNA cleavage sites detected in nanoPARE data sets
**Supplemental Data Set 4.** Normalized transcript levels in wild-type and miRNA-deficient *dcl1*-*5* globular embryos

## DIVE Curated Terms

The following phenotypic, genotypic, and functional terms are of significance to the work described in this paper:

SYP122 Gramene: AT3G52400
SYP122 Araport: AT3G52400

mir160a Gramene: AT2G39175
mir160a Araport: AT2G39175

EIF4A1 Gramene: AT3G13920
EIF4A1 Araport: AT3G13920

WOX2 Gramene: AT5G59340
WOX2 Araport: AT5G59340

AGO2 Gramene: AT1G31280
AGO2 Araport: AT1G31280

DCL1 Gramene: AT1G01040
DCL1 Araport: AT1G01040
